# Analysis of Doppler Effect on the Pulse Compression of Different Codes Emitted by an Ultrasonic LPS

**DOI:** 10.3390/s111110765

**Published:** 2011-11-15

**Authors:** José A. Paredes, Teodoro Aguilera, Fernando J. Álvarez, Jesús Lozano, Jorge Morera

**Affiliations:** Sensory Systems Research Group, University of Extremadura, 06006 Badajoz, Spain; E-Mails: teoaguibe@unex.es (T.A.); fafranco@unex.es (F.J.Á.); jesuslozano@unex.es (J.L.); george@unex.es (J.M.)

**Keywords:** doppler shift, ultrasonic LPS, kasami sequences, complementary set of sequences, loosely synchronous sequences

## Abstract

This work analyses the effect of the receiver movement on the detection by pulse compression of different families of codes characterizing the emissions of an Ultrasonic Local Positioning System. Three families of codes have been compared: Kasami, Complementary Sets of Sequences and Loosely Synchronous, considering in all cases three different lengths close to 64, 256 and 1,024 bits. This comparison is first carried out by using a system model in order to obtain a set of results that are then experimentally validated with the help of an electric slider that provides radial speeds up to 2 m/s. The performance of the codes under analysis has been characterized by means of the auto-correlation and cross-correlation bounds. The results derived from this study should be of interest to anyone performing matched filtering of ultrasonic signals with a moving emitter/receiver.

## 1. Introduction

Local Positioning Systems (LPS), intended to locate people and/or objects in indoor environments, constitute one of the core elements of the so-called Intelligent Environments (IE). The interest to develop this type of systems has significantly grown in the last years, with the appearance of proposals based on different technologies that include ultrasonic [1], magnetic [2], optic [3] and radiofrequency [4]. Among them, ultrasound represents a classic solution whose popularity comes from its low cost and high reliability.

Yet during the first years of the former decade, some systems were proposed that achieved centimetric precision through the emission of ultrasonic pulses, both centralized, where the object to be located acts as the emitter [1], and localized, where this object is in charge of computing its own position using the signals received from different beacons [5]. Shortly after, signal coding was incorporated into these systems, choosing for this purpose families of binary codes with good correlation properties. This improvement brought important advantages: simultaneous emission, higher precision, larger robustness to noise and the capability to introduce privacy in the location process. Since one of the first encoding proposals carried out by Hazas and Ward with Gold sequences [6], several works have arisen in the field of acoustic LPS that employ more and more complex encoding schemes, with the emission of Kasami sequences [7], Complementary Sets of Sequences (CSS) [8] and Loosely Synchronous (LS) codes [9].

Many authors have already pointed out the problems that the receiver movement could cause on the detection of ultrasonic encoded signals, since the Doppler effect undergone by these signals could make them completely unrecognizable to the matched filter installed in the receiver [10,11]. In a previous work, the authors presented a complete software model for an ultrasonic LPS that could be used to simulate this effect [12], and it was shown that radial speeds as low as 1.5 m/s could drastically deteriorate the autocorrelation properties of the signals emitted by this particular system: 255-bit Kasami sequences with BPSK modulation at 50 kHz. More recently, a detailed analysis of this phenomenon has been presented by quantifying the increment of the auto- and cross-correlation bounds of four different lengths of Kasami sequences with increasing speed of the receiver [13,14]. Also the shift of the autocorrelation peak was studied in these works, which showed that this effect is not very important in practice. The reason is that long before this shift increases more than a few sampling periods, the auto-correlation bound rises above the detection threshold.

This work represents a significant extension of the work carried out by the authors in those papers, since three different families of binary codes are compared in terms of their bound values when detected by pulse compression with a moving receiver. The analysis is first performed with a high versatility simulator that allows the user to choose among different families of codes and modulation schemes, as well as to model the effect of various phenomena that characterize the propagation of ultrasonic signals in air: geometrical spreading, atmospheric absorption and the filtering associated with the transducers. Next, the results obtained with this simulator for a particular family of codes and modulation scheme are experimentally validated making use of an electric slider with which the speed of the ultrasonic emitter/receiver can be accurately controlled. These results are finally discussed in the last section, where the main conclusions of this work are also outlined.

## 2. Emission Features

As stated before, three different codes already used in the design of ultrasonic LPS are compared in this work. The main features of these codes are briefly described in this section, where also the modulation scheme and the parameters used to characterize the comparative analysis are presented.

### 2.1. Binary Codes

#### 2.1.1. Kasami Sequences

Kasami sequences [15] belong to the well-known family of Pseudo Noise (PN) Sequences, that are generated using linear feedback shift-registers and exclusive OR-gate circuits. A new Kasami sequence *k*[*n*] can be obtained from a maximal sequence and the decimated and concatenated version of this sequence by performing the module-2 sum of the former with any delayed version of the latter, *i.e.*,

(1) $$k=m_{1}⊕D^{l}m_{2}\;\;\;\text{with}\;l<L$$

where *m*_1_ is a maximal sequence of length *L* = 2^*N*−1^ with *N* even, *m*_2_ is the sequence obtained from the decimation of *m*_1_ with a decimation factor of *q* = 2^*N*/2^ + 1 and the concatenation of the result *q* times, ⊕ represents the module-2 sum and *D^l^m*_2_ is the sequence obtained by cyclically shifting *l* positions the *m*_2_ sequence. Three different families of four sequences with lengths of 63, 255 and 1,023 bits have been generated following this procedure. With this number of sequences in a family, a usual 4-beacon LPS architecture is being assumed in this work.

#### 2.1.2. Complementary Set of Sequences (CSS)

A set of *p* binary sequences {*x_i_*[*n*], 1 ≤ *i* ≤ *p*} whose elements are either +1 or −1 is a complementary set if the sum of their aperiodic auto-correlation functions *ϕ_xixi_* equals zero for all nonzero time shifts, *i.e.*,

(2) $$\phi_{x1x1}\left[\tau \right]+\phi_{x2x2}\left[\tau \right]+\cdots +\phi_{\mathrm{xpxp}}\left[\tau \right]=\{\begin{array}{ll} p\cdot L & \tau =0\\ 0 & \tau \neq 0 \end{array}$$

where *L* is the length of the sequences. Two sets with the same number of sequences {*x_i_*[*n*], *y_i_*[*n*]; 1 ≤ *i* ≤ *p*} are said to be orthogonal when the sum of the corresponding cross-correlation functions equals zero, *i.e.*,

(3) $$\phi_{x1y1}\left[\tau \right]+\phi_{x2y2}\left[\tau \right]+\cdots +\phi_{\mathrm{xpyp}}\left[\tau \right]=0\;\;\;\;\forall \tau$$

Complementary sets of more than two sequences were initially studied by Tseng and Liu [16], who gave a general method for constructing sets of longer sequences from shorter ones. Based on this method a new algorithm was proposed to recursively generate complementary sequences with power-of-four length starting from the elementary delta sequences [17]. This algorithm has been employed in this work to generate three families of four sets, each set composed of four sequences {*x*_1_, *x*_2_, *x*_3_, *x*_4_} with lengths of 16, 64 and 256 bits. The four sequences composing a set are finally interleaved to generate three different emission sequences *c*[*n*] of 64, 256 and 1,024 bits as follows:

(4) $$c\left[n\right]=\left[x_{1}\left[1\right]x_{2}\left[1\right]x_{3}\left[1\right]x_{4}\left[1\right]\cdots x_{1}\left[L\right]x_{2}\left[L\right]x_{3}\left[L\right]x_{4}\left[L\right]\right]$$

#### 2.1.3. Loosely Synchronized (LS) Sequences

LS codes can be generated from Golay [18,19] or CSS [20] codes, and they exhibit an Interference Free Window (IFW), where the aperiodic auto-correlation sidelobes and cross-correlation values become zero. The total length of these codes can be expressed as *L* = *P · N* + *W*_0_, where *P* is the number of codes with orthogonal properties in the IFW, *N* is the length of the Golay pair the LS sequences are generated from, and *W*_0_ is the number of zeros inserted in the center of the LS code, that determines the total length of the IFW as min(2*N* − 1, 2*W*_0_ + 1) (*W*_0_ is usually chosen as *N* − 1). If a family of *K* LS codes with length *L* is represented as {*l_k_*[*n*]; 1 ≤ *k* ≤ *K*;0 ≤ *n* ≤ *L* − 1}, the correlation properties described above can be expressed as,

(5) $$\phi_{l_{i}l_{j}}\left[\tau \right]=\{\begin{array}{lll} K\cdot N, & \text{for}\;\tau =0, & i=j\\ 0, & \text{for}\;1\leq \left|\tau \right|\leq W_{0}, & i=j\\ 0, & \text{for}\;0\leq \left|\tau \right|\leq W_{0}, & i\neq j \end{array}$$

where *ϕ_xy_* is again the aperiodic correlation between sequences *x* and *y*. When using LS sequences, not only the Multiple-Access-Interference (MAI) due to the non-zero cross-correlation values, but also the Inter-Symbol-Interference (ISI) due to the non-zero value of the auto-correlation sidelobes are ideally eliminated provided the maximum transmission delay is less than half the IFW (*W*_0_). Three families of four LS codes with lengths of 71, 271 and 1,087 bits have been generated in this work from Golay codes of 8, 16 and 64 bits respectively, following the procedure proposed in [18].

### 2.2. Modulation Scheme

**Figure 1.**
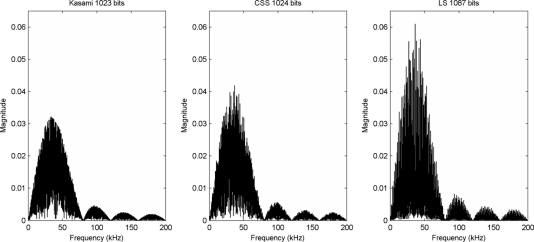
Spectral features of the longest BPSK modulated codes.

In order to adapt the spectral features of the emissions to the frequency response of the ultrasonic transducer, these codes are binary phase modulated (BPSK). This modulation scheme has been widely used to transmit binary codes in matched filtering-based sonar systems. Every bit in the code *q*[*n*] is modulated with one or more carrier cycles whose phase, 0 or π, is given by the bit value to obtain the modulated pattern as:

(6) $$p\left[n\right]=\sum_{i=0}^{L-1}q\left[i\right]\cdot m\left[n-i\cdot M\right]$$

where *L* is the code length, m[*n*] is the modulation symbol, and *M* represents the number of samples in this symbol, given by the product between the number of carrier cycles composing this symbol and the ratio between the sampling and the carrier frequencies. Figure 1 shows the spectral features of the BPSK modulated versions of the longest codes described in the previous section when using a modulation symbol of one 40 kHz-carrier cycle and a sampling frequency of 400 kHz, *i.e.*,

(7) $$m\left[n\right]=\{\begin{array}{ll} \text{sin}(0.2\pi \cdot n) & n\in \left\{0,\ldots ,9\right\}\\ 0 & \text{otherwise} \end{array}$$

### 2.3. Parameters Under Analysis

A common measure for the performance of a family of *K* modulated codes is given by the auto-correlation (AC) and cross-correlation (CC) bounds, defined as:

(8a) $$\theta_{\mathrm{AC}}=\text{max}\;\left\{\begin{array}{cc} \frac{\phi_{s_{i}p_{i}}\left[k\right]\forall k\notin \left[-G\cdot M,G\cdot M\right]}{\text{max}\phi_{s_{i}p_{i}}} & \forall i\in \left\{1,\ldots ,K\right\} \end{array}\right\}$$

(8b) $$\theta_{\mathrm{CC}}=\text{max}\;\left\{\begin{array}{ccc} \frac{\phi_{s_{i}p_{j}}\left[k\right]\forall k}{\text{max}\phi_{s_{i}p_{i}}} & \forall i,j\in \left\{1,\ldots ,K\right\}, & i\neq j \end{array}\right\}$$

where *ϕ_sp_*[*k*] is the aperiodic correlation function between the received signal *s* and the stored pattern *p*, *M* is again the number of samples in the modulation symbol, and *G* is a guard factor whose value is chosen between 1 and 5 (3 in this work). The first bound will give a measure of the difficulty to detect a code received at a certain speed, whereas the second bound will show how the cross-correlation properties of the family are deteriorated as a consequence of this movement. Depending on the application, the upper threshold for these magnitudes are typically in the range 0.3–0.5.

These parameters have to be slightly modified when dealing with LS sequences, since only the correlation values obtained inside the IFW are of interest in this case. Taking into account that this window is limited by two correlation peaks with half the height of the main AC peak, and to avoid the effects derived from the correlation of the modulation symbol, the new bounds are defined as:

(9a) $$\theta_{\mathrm{AC},\mathrm{LS}}=\text{max}\;\left\{\begin{array}{cc} \frac{\phi_{s_{i}p_{i}}\left[k\right]\forall k\in \left[-W_{0}+G\cdot M,-G\cdot M\right]\cup \left[G\cdot M,W_{0}-G\cdot M\right]}{\text{max}\;\phi_{s_{i}\;p_{i}}} & \forall i\in \left\{1,\ldots ,K\right\} \end{array}\right\}$$

(9b) $$\theta_{\mathrm{CC},\mathrm{LS}}=\text{max}\;\left\{\begin{array}{cc} \frac{\phi_{s_{i}\;p_{j}}\left[k\right]\forall k\in \left[-W_{0}+G\cdot M,W_{0}-G\cdot M\right]}{\text{max}\phi_{s_{i}p_{i}}} & \forall i,\;j\in \left\{1,\ldots ,K\right\},\;\;i\neq j \end{array}\right\}$$

where *W*_0_ is half the size of the IFW as defined in Section 2.1.3.

## 3. Simulated Model

### 3.1. Ideal Results

This section shows the results obtained by the simulator when only the effect of the receiver movement is modeled, and no further phenomena characterizing a particular system are considered. This effect can be easily simulated by assuming a virtual sampling frequency *f′_s_* for the emitted signal, given by:

(10) $$f_{s}^{\prime }=f_{s}\left[1-\frac{{\vec{v}}_{r}}{c}\frac{{\vec{r}}_{r}-{\vec{r}}_{e}}{\left|{\vec{r}}_{r}-{\vec{r}}_{e}\right|}\right]$$

where *f_s_* is the actual sampling frequency, c *≃* 343 m/s is the sound speed in air at a temperature of 20 C and pressure of 1 atm, *r⃗_r_* is the receiver position vector, *r⃗_e_* is the emitter position vector, and *v⃗_r_* is the receiver velocity vector. From this frequency, the signal acquired by the receiver at the actual sampling frequency is obtained by cubic spline interpolation [21].

**Figure 2.**
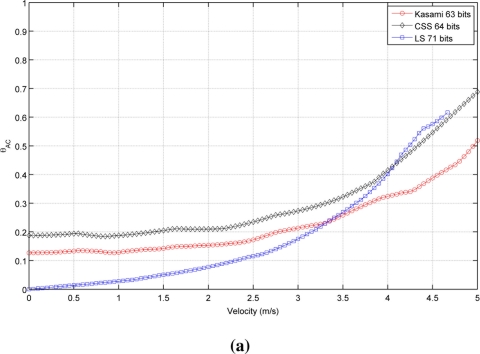

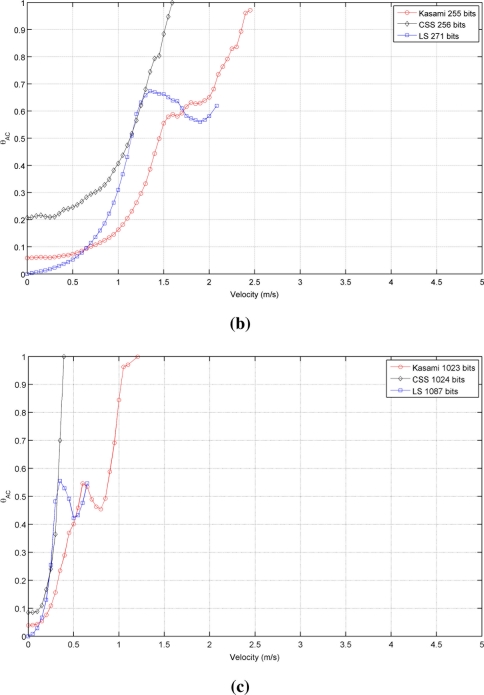
Simulated auto-correlation bound for the sequences with lengths close to **(a)** 64 bits **(b)** 256 bits **(c)** 1,024 bits.

Figure 2 shows the autocorrelation bound θ*_AC_* as a function of the receiver velocity for the sequences with lengths close to (a) 64, (b) 256 and (c) 1,024 bits. As can be seen, in all cases, the lowest value with a static receiver (*v⃗_r_* = 0) is provided by LS sequences, followed by Kasami and finally by CSS, that exhibit the worst behavior. Note that, although the initial values of θ*_AC_* are better for LS sequences, the increment of its value with receiver velocity is slower for Kasami and both curves cross at a certain point. Also, note that the curve representing the behavior of LS sequences never reaches bound values close to 1 and always ends with a value in between 0.6 and 0.7. This is a negative consequence of the lateral peaks appearing at the boundaries of the IFW, and one of the main drawbacks of LS sequences against the other families of binary codes. Long before the auto-correlation bound can reach values close to 1, these lateral peaks can take amplitudes larger than that of the main peak, thus confusing the peak detector.

Table 1 shows the receiver velocity for which θ*_AC_* reaches the practical limit value of 0.5. As can be seen, Kasami sequences provide the larger range of admissible velocities in all cases.

As pointed out in the Introduction, one of the main advantages of encoding the ultrasonic signals of a LPS is the capability to perform simultaneous emission of all beacons whose signals will be distinguished by the receiver despite their possible overlapping. For this reason, it is important to analyze the effect of the receiver movement not only in the auto-correlation of the emitted codes but also in the cross-correlations between all the codes in the family. As stated before, we have supposed that our LPS is composed of four beacons performing the simultaneous emission of different codes with good correlation properties, and thus, families of four members (codes) have been generated.

**Figure 3.**
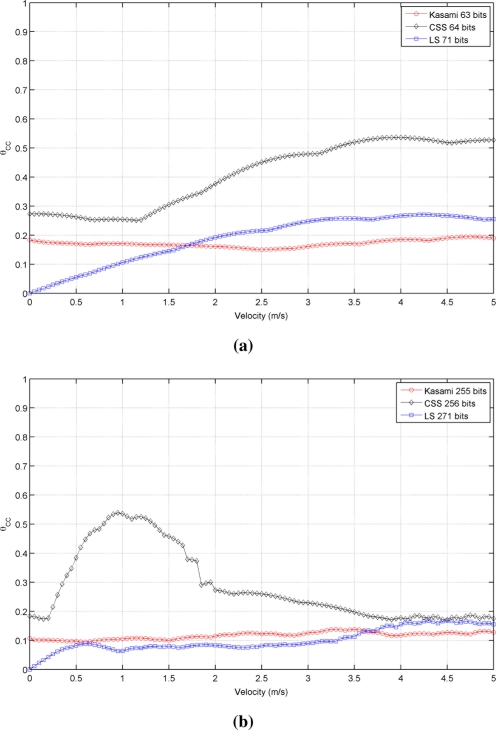

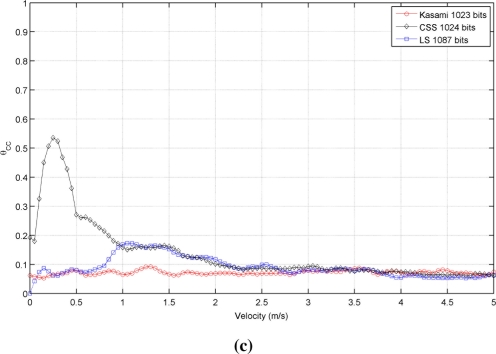
Simulated cross-correlation bound for the sequences with lengths close to **(a)** 64 bits **(b)** 256 bits **(c)** 1,024 bits.

Figure 3 shows the simulated results obtained for the cross-correlation bound θ*_CC_* as a function of the receiver velocity, for all lengths and sequences under consideration. This figure shows that the receiver movement does not significatively worsen the cross-correlation properties of Kasami codes with respect to the values obtained with a stationary receiver. The behavior of the other two sequences is slightly different. CSS codes begin with a low cross-correlation bound, then, a range of velocities where this bound increases significantly appear, and finally, it decreases to a practically constant value. This tendency is repeated for all lengths, although the range of velocities with large θ*_CC_* values become narrower with longer sequences. Finally, LS sequences exhibit zero θ*_CC_* values at zero velocity, and then, this bound increases showing certain variability. In all cases, the best cross-correlation values at high velocities are provided by Kasami codes, although the differences among the three sequences under consideration are not significant.

It is important to remark that the families of binary codes chosen to conduct the study presented in this work are those with the best correlation properties among all the families that can be generated from the same algorithms with the same number of members and lengths. If other families are used, similar trends to those observed in Table 1 and Figures 2 and 3 will be obtained, but with slightly increased values of bound.

### 3.2. Transducer Response and Atmospheric Absorption Modeling

Prior to obtaining experimental data with the equipment that will be described in Section 4, two phenomena characterizing this particular experimentation must be included in the model employed in Section 3.1, since they could have a strong influence on the simulated results. These phenomena are:

1. The frequency response of the ultrasonic transducer, with a nominal bandwidth of 10 kHz at −6 dB according to the manufacturer.
2. Atmospheric absorption of ultrasound in air at the laboratory temperature and humidity conditions. This absorption coefficient is strongly dependent on frequency.

**Figure 4.**
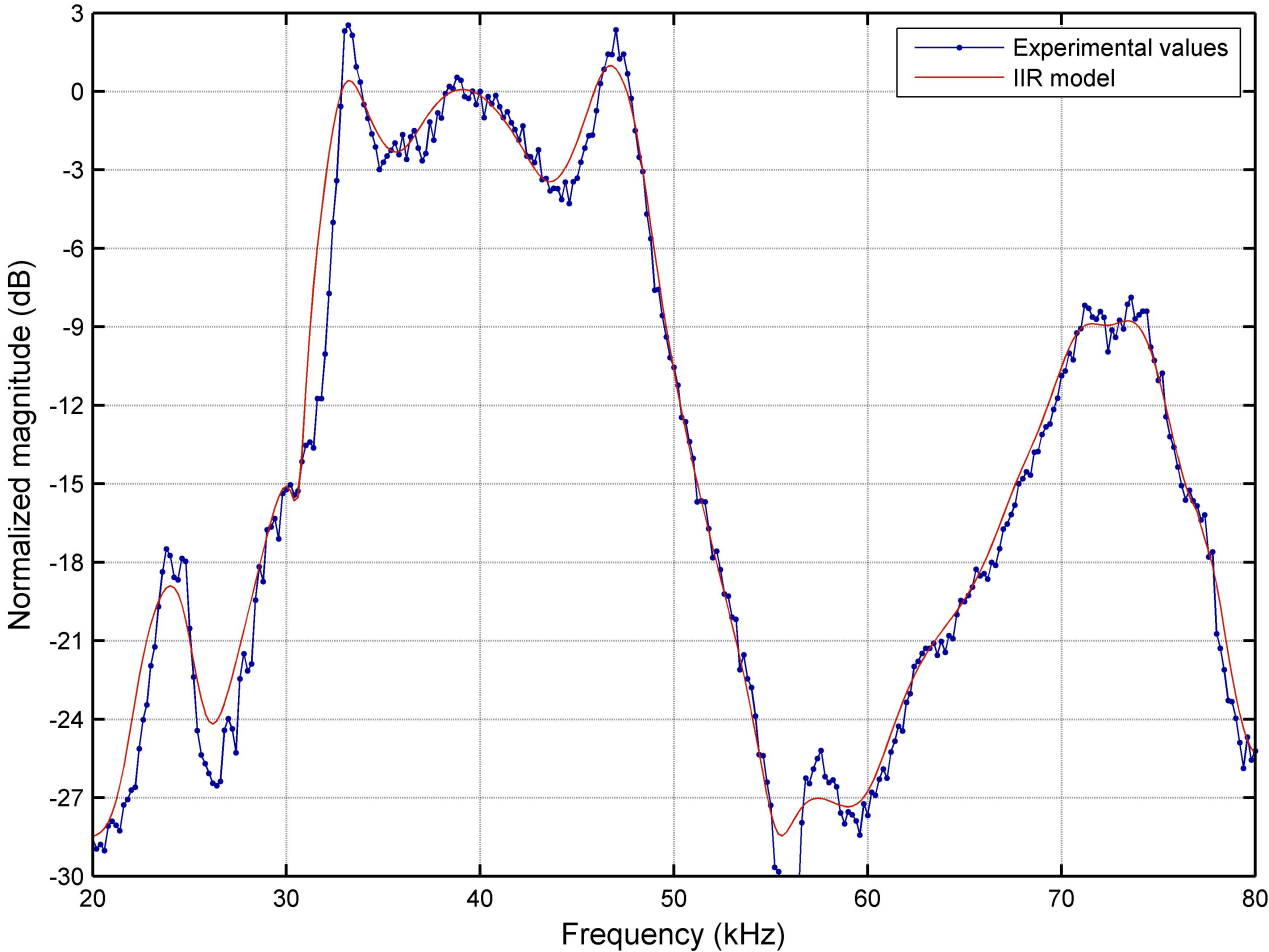
Emitter frequency response: experimental values (blue dotted) and IIR filter model (red solid).

To model the first phenomenon, an accurate experimental analysis of the frequency response of the the emitter (driver + transducer) has been carried out in the range 20–80 kHz, obtaining the results shown in Figure 4 (blue dotted line). This response has been modeled with a 50 order IIR filter whose frequency response is also represented in Figure 4 with a red solid line. As can be seen in this figure, the actual −6 dB bandwidth experimentally measured with respect to the reference frequency of 40 kHz is about 16.5 kHz.

With respect to the atmospheric absorption of ultrasound in air, it has been modeled as dictated by the ISO 9613-1 (1993) standard [22]. To emulate the laboratory conditions, a temperature of 20 °C, a relative humidity of 60% and an atmospheric pressure of 1 atm have been assumed. Also, an average separation distance of 1 m between the emitter and the receiver has been considered. In all cases, we have observed that the variations taking this phenomenon into account are negligible. A new set of simulations have proved that the frequency dependence of the absorption coefficient would start distorting the received signal for a emitter-receiver distance of about 20 m, a distance that is unlikely to be usable in practice, given the reduced Sound Pressure Level (SPL) of our emitter.

### 3.3. New Simulated Results

**Figure 5.**
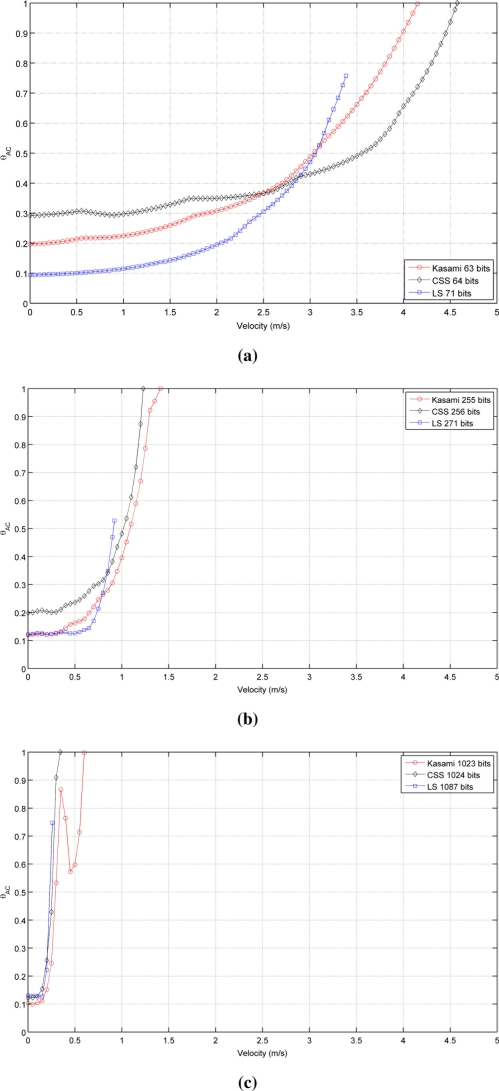
Simulated auto-correlation bound for the sequences with lengths close to **(a)** 64 bits **(b)** 256 bits **(c)** 1,024 bits (transducer response and atmospheric absorption are considered).

**Figure 6.**
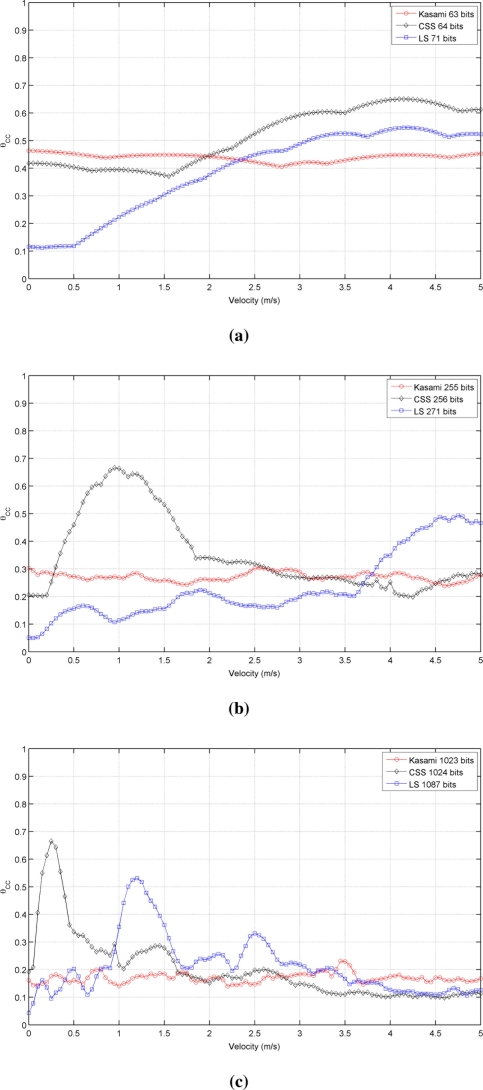
Simulated cross-correlation bound for the sequences with lengths close to **(a)** 64 bits **(b)** 256 bits **(c)** 1,024 bits (transducer response and atmospheric absorption are considered)

Figure 5 shows the behavior of the auto-correlation bound for the same cases represented in Figure 2, but taking now into account the phenomena described before. As can be seen, all curves present a similar trend but with increased values of θ*_AC_*, thus reducing the valid range of velocities that assure values of θ*_AC_* ⩽ 0.5, as shown in Table 2. Kasami sequences are still the best choice with long families, although the distance between these sequences and CSS has been reduced. Also, note that the auto-correlation bound of LS sequences with a static receiver is no longer zero. Similar conclusions can be drawn from the comparison of Figures 3 and 6, this latter including the modeling of the transducer response and atmospheric absorption. Shorter Kasami sequences seem to be the more sensitive codes to these phenomena.

## 4. Experimental Analysis and Results

**Figure 7.**
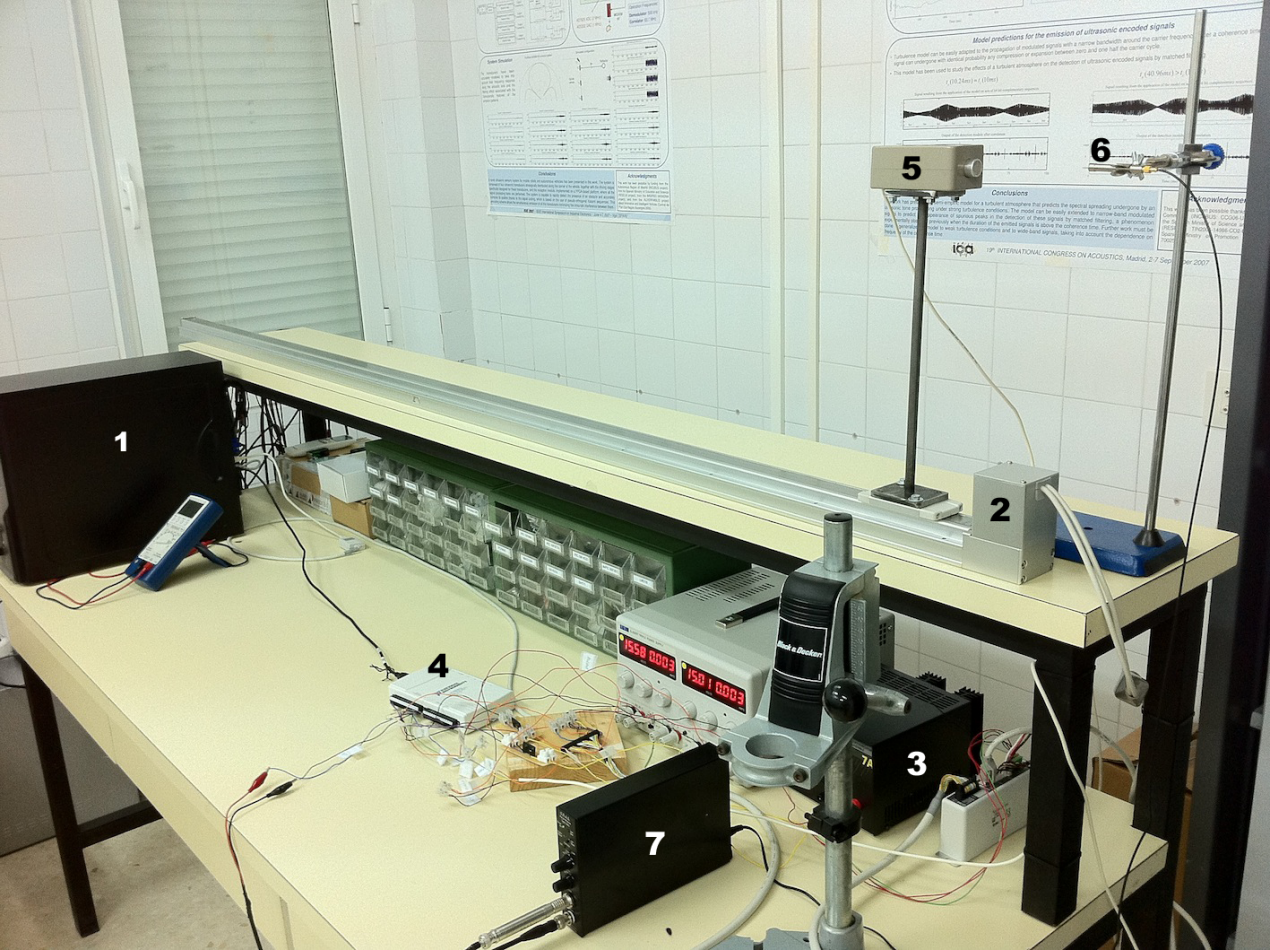
Experimental setup.

This section presents the experimental analysis carried out to validate the simulated results obtained in the previous section. A picture of the experimental equipment employed in this analysis is shown in Figure 7. This equipment is composed of:

1. Computer: from where a software application controls the emission, the movement of the platform supporting the emitter, and the acquisition parameters. The received data are stored in a text file for their latter processing.
2. Electric slider: two meters long conveyor belt where the ultrasonic transducer is fixed. A rigid metallic platform has been built to separate the emitter from the base and avoid undesired echoes. This slider is capable to reach a maximum speed of 2 m/s, with maximum acceleration values of ±3 m/s^2^, thus providing an analysis window of about 800 ms of constant velocity.
3. Two DC sources: one providing 24 V and 7 A for the electric slider, and the second providing 24 V and 3 A for the controller.
4. NI USB-6212 data acquisition card, to transmit the modulated code to the emitter and acquire the signal received by the microphone at a sampling frequency of 400 kS/s.
5. Prowave 400WB160 ceramic ultrasonic transducer, with a central operation frequency of 40.0 ± 1.0 kHz, a nominal bandwidth of 10 kHz at −6 dB, and a SPL of 105 dB min with respect to 20 μPa at 30 cm. A driver module has been specifically designed for this transducer, based on a TL082 operational amplifier in inverting configuration, providing a gain of −3 V/V.
6. G.R.A.S. 40BE free-field pre-polarized ultrasonic microphone, with a sensitivity of 4 mV/Pa, a dynamic range of 3—166 dB with respect to 20 μPa, and a flat frequency response in the range 4 Hz–100 kHz.
7. G.R.A.S. 12AK power module, that provides a signal amplification of 40 dB in the range of frequencies of interest and performs a high-pass filtering with a cutoff frequency of 20 Hz.

In order to test the functionality of this equipment, a proof 40 kHz carrier signal has been continuously emitted during the following 4-stage trajectory:

1. Acceleration at 3 m/s^2^ for 666 ms, until the maximum nominal speed of 2 m/s is reached.
2. Constant speed movement at 2 m/s for 300 ms.
3. Deceleration at −3 m/s^2^ until stop.
4. Static emission until a total emission time of 2 s is completed.

**Figure 8.**
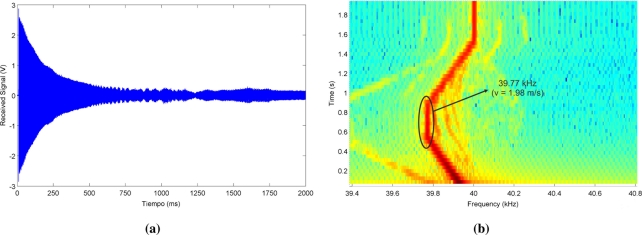
Features of the proof 40 kHz carrier signal. **(a)** Received Signal; **(b)** Spectrogram.

Figure 8**(a)** shows the actual signal received by the microphone during this trajectory, and Figure 8**(b)** represents the spectrogram of this signal, where the Doppler shift associated to the different movements of the emitter can be clearly visualized. A detailed spectral analysis of the signal received during the second stage reveals a central frequency of 39.77 kHz, *i.e.*, a Doppler shift of 0.23 kHz that corresponds to a emitter speed of 1.98 m/s if a sound speed of 343 m/s is considered.

**Figure 9.**
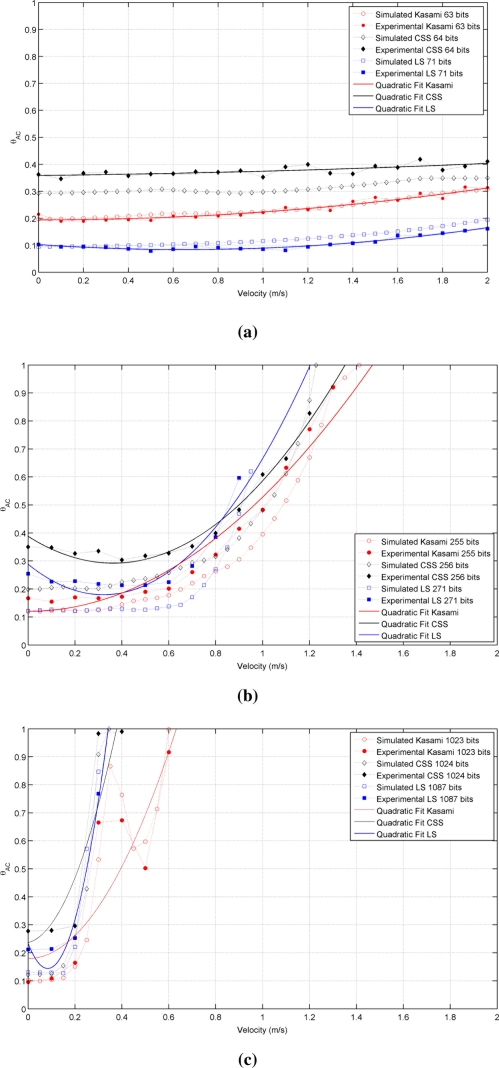
Simulated and experimental auto-correlation bounds for the sequences with lengths close to **(a)** 64 bits **(b)** 256 bits **(c)** 1,024 bits.

The experimental analysis has been carried out by increasing the emitter velocity from 0 to 2 m/s in steps of 0.1 m/s for all sequences and code lengths under consideration. During each trial, every code is emitted once when the platform has reached the constant velocity regime, and these trials are repeated ten times to provide average values of the auto- and cross-correlation bounds. Figure 9 shows the actual results obtained for the autocorrelation bound in all cases, together with the simulated results obtained in Section 3.3 for this range of velocities. As shown, there is a good agreement between both sets of data in all cases. The experimental values have been fitted to a second order polynomial in the form θ*_AC_*(*v*) = A + B *v* + C *v*^2^, that is also represented in this figure as a continuous line. This quadratic fit, whose coefficients are presented in Table 3 for all sequences under analysis, is a useful tool to estimate the expected value of θ*_AC_* for a particular sequence and a particular receiver speed.

**Figure 10.**
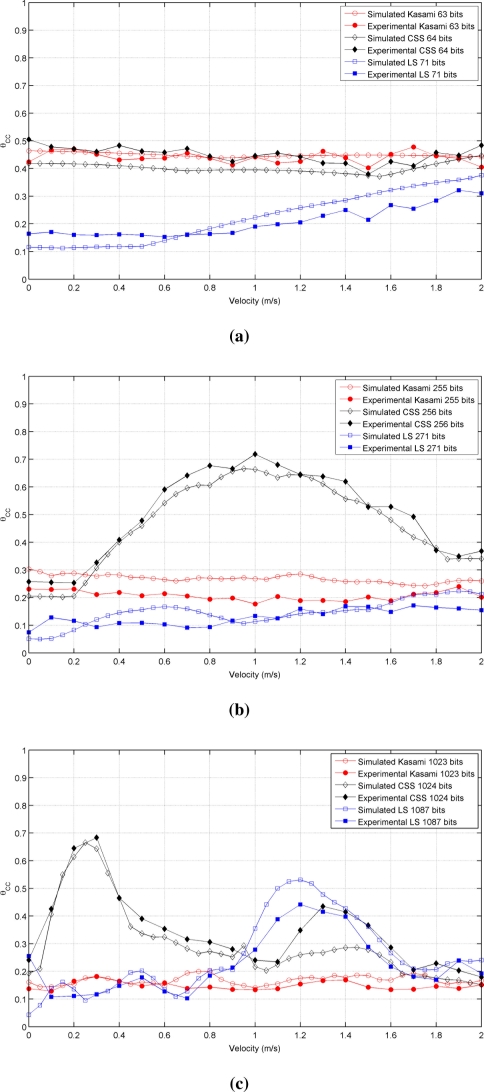
Simulated and experimental cross-correlation bounds for the sequences with lengths close to **(a)** 64 bits **(b)** 256 bits **(c)** 1,024 bits.

Finally, Figure 10 shows the actual results obtained for the cross-correlation bound in all cases, together with the simulated results presented in Section 3.3 for this parameter. Again, good agreement is observed between simulated and experimental results.

## 5. Discussion and Conclusions

This work has presented a detailed study of the influence that the receiver velocity can have on the matched filtering of the signals emitted by a particular ultrasonic LPS. Families of four BPSK modulated Kasami, LS and CSS sequences with different lengths have been considered in this study, establishing for each one of them the range of admissible receiver velocities in terms of the auto-correlation and cross-correlation bounds of the corresponding family. These results have been experimentally validated with the help of an electric slider, and these experimental data have been fitted to a second order polynomial that can be used to easily determine the worsening of the bound as a function of the receiver velocity.

Both the simulated and the experimental results confirm that Complementary Set of Sequences are not the best choice when dealing with a moving receiver, since the auto-correlation bound of this family has a high value over the entire range of velocities. Also, the cross-correlation bound of this family exhibits a range of velocities where it increases significantly with respect to its static value. As expected, LS codes present the lowest values of auto- and cross-correlation bounds with a static receiver for all lengths, although these values increase with the receiver velocity exceeding those obtained with Kasami sequences at a certain point. Furthermore, the range of admissible velocities is reduced in this case due to the presence of large sidepeaks at the bounds of the Interference Free Window, peaks that can exceed the main peak of the auto-correlation long before the bound reaches values close to 1.

Kasami sequences seem to be a good choice when matched filtering of ultrasonic signals is used with a moving emitter/receiver. Although the static value of their auto-correlation bound is greater than that of LS sequences, the worsening of this bound with increasing receiver velocity is relatively slow, thus providing the largest range of admissible velocities. Also, conversely to the behavior obtained with the other families of binary codes, the cross-correlation bound of Kasami sequences remains at a relatively constant value throughout all the range of velocities analyzed.

Some work is still to be done in this interesting field of research. For example, only one modulation scheme has been considered in our study (BPSK), but other possibilities should be explored. Even more interesting, new encoding sequences are currently being developed with promising performances against Doppler shift, such as the use of Multilevel Complementary Sequences [23]. These sequences should be subject of further analysis.

## Figures and Tables

**Table 1.** Receiver velocities (m/s) whose θ*_AC_* is 0.5 (ideal simulation).

	**Kasami**	**LS**	**CSS**
∼ 64 bits	4.9	4.2	4.3
∼ 256 bits	1.4	1.1	1.1
∼ 1,024 bits	0.6	0.3	0.3

**Table 2.** Receiver velocities (m/s) whose θ*_AC_* is 0.5 (transducer response and atmospheric absorption are considered).

	**Kasami**	**LS**	**CSS**
∼ 64 bits	3.0	3.0	3.5
∼ 256 bits	1.0	0.9	1.0
∼ 1,024 bits	0.3	0.2	0.3

**Table 3.** Polynomial coefficients of the quadratic fit for all sequences.

**Sequence**	**Length**	**A**	**B**	**C**
Kasami	63 bits	0.1941	−0.0007	0.0299
	255 bits	0.1200	0	0.4083
	1,023 bits	0.1796	0	2.0547

LS	71 bits	0.1026	−0.0578	0.0446
	271 bits	0.2882	−0.6773	1.0550
	1,087 bits	0.2340	−2.1490	12.8500

CSS	64 bits	0.3588	0.0081	0.0067
	256 bits	0.3879	−0.5263	0.7246
	1,024 bits	0.2371	0.1860	4.8540
